# Dynamic causal models in infectious disease epidemiology—an assessment of their predictive validity based on the COVID-19 epidemic in the UK 2020 to 2024

**DOI:** 10.3389/fpubh.2025.1573783

**Published:** 2025-05-02

**Authors:** Cam Bowie, Karl Friston

**Affiliations:** 1 Retired, Devon, United Kingdom; 2 Queen Square Institute of Neurology, University College London, London, United Kingdom

**Keywords:** coronavirus, compartmental models, epidemiology-descriptive, incidence, public health methodology, dynamic causal modeling

## Abstract

This technical report addresses the predictive validity of long-term epidemiological forecasting based upon dynamic causal modeling. It uses complementary prospective and retrospective analyses. The prospective analysis completes a series of (annual) reports comparing predictions with subsequent outcomes (i.e., cases, deaths, hospital admissions and Long COVID) reported a year later. Predictive validity is then addressed retrospectively by examining predictions at various points during the pandemic, in relation to actual outcomes at three, six and 12 months after the predictions were evaluated. This analysis suggests that—with a sufficiently expressive dynamic causal model—three, six and 12 month projections can be remarkably accurate (to within 10% or less of observed outcomes) at certain phases of the epidemic: namely, the initial phase—before the emergence of highly transmissible variants—and toward the end of the pandemic, when slow fluctuations in transmissibility and virulence can be estimated more precisely. However, the predictive accuracy in the intervening periods are compromised, to the extent that some forecasts only remain within their Bayesian credible intervals for 3 months. We provide a quantitative analysis of predictive accuracy for future reference and discuss the implications for epidemiological modeling, and forecasting, of this sort.

## Introduction

A Dynamic Causal Model (DCM) of the COVID-19 outbreak in the UK in February 2020 (1) was used to provide long-term forecasts—on a fortnightly basis—from February 2021 to November 2023 (2). Published reports using the DCM have examined the epidemic in different countries and regions, various mitigation strategies and the reliability of projections of the model in peer reviewed journals (3–10) and pre-prints (11–18). As promised—in a previous analysis of the predictive validity of long term projections (8)—this technical report assesses a further 12 month projection from November 2023 to October 2024 in the UK. It then presents a retrospective analysis of the predictive accuracy at 3, 6, and 12 months, at yearly intervals during the pandemic.

We first summarize a prospective analysis of predictive validity for the period covering November 2023 to October 2024. This complements analyses of previous years and reaffirms the predictive accuracy of certain projections; particularly the incidence of cases, Long COVID and hospital admissions. Although earlier projections of COVID-related deaths were underestimated by a factor of two, predictions for the last year were accurate to within 20%. These analyses are then repeated retrospectively, to assess the predictive accuracy of DCM in terms of projections a few months into the future—based upon retrospective data—throughout the pandemic.

## Outcomes for November 2023 to October 2024

### Methods and data

Since we published our projections for the year to October 2024 some of the data sources have changed or have been deprecated. The Institute for Health Metrics and Evaluation (IHME)—which we had used as a comparison—stopped estimating incidence in April 2023 (19). The Office of National Statistics (ONS)—in conjunction with the UK Health Security Agency (UKHSA) set up in October 2023—reported on a Winter Covid Infection Survey in April 2024 (20). The UKHSA have published a pre-print of estimates of incidence using this survey data (21). While their estimates cover only the winter months, graphs of incidence can be compared to our incidence graph for the same period.

The only COVID-19 data published by UKHSA up to October 2024 on testing are the number of people receiving a PCR test and the positivity of people receiving such a PCR test (22). While we published our projection to October 2024 of PCR and LFT tests combined we also estimated (but did not report) our projection of PCR tests alone, which we refer to in this report. We also separate out the PCR positive tests from confirmed cases by both PCR tests and LFTs to provide a fair comparison.

UKHSA also stopped reporting deaths within 28 days of a positive PCR test in July 2023 (23). The UKHSA have assessed the comparability of death registrations with this metric and found that in the second half of 2022 about 40% of deaths within 28 days of a positive test where registered deaths involving COVID-19 and 30% being caused by COVID-19 (24). As with testing, we estimated projections of registered deaths as well as registered deaths within 28 days of a test in November 2023 and provide these results.

At the time of writing, hospital admissions are still being provided on the UKHSA dashboard. The Winter Covid Infection Survey has published estimates of post COVID-19 syndrome (Long COVID of more than 12 weeks) UK prevalence and estimates of duration (20). These can be used to calculate incidence which can be used to compare with our projections. The model used in November 2023 modified a test and trace parameter from 0.29 to 0.25 on 1st October 2023. This model was used to estimate projected values to September 2024.

### Results

Table 1 of our previous report (8) is provided to show our projections (in the far right column) of the year to October 2024 of cumulative results provided by the Nov2023 model.

**Table 1:** Cumulative numbers of COVID-19 cases, deaths, tests, hospital admissions and post COVID-19 syndrome—1st February 2020—1st October 2023 and 12 month projected numbers for 1st October 2023–2024—UK with projection using the November 2023 model.

Scenario assuming FTTIS is 25% effective	DCM 2022 projection	Actual	Data source	DCM 2023 projection
Cumulative totals from 1st February 2020 to	1st October 2023	1st October 2023		1st October 2023 to 1st October 2024
Estimated incidence	485,603,813	131,242,140	IHME—1 Apr 2023	40,692,662
Confirmed cases by PCR and LFT	53,409,837	24,743,787	Our World in Data—30 Sep 2023	524,351
Deaths within 28 days of a positive PCR test	330,957	229,765	Our World in Data—30 Sep 2023	24,100
Tests (both PCR and LFD)	821,181,901	602,512,524	UK Covid-19 dashboard—30 Sep 2023	14,080,675
Hospital admissions	1,867,580	862,553	UK Covid-19 dashboard—30 Sep 2023	175,303
Post Covid-19 Syndrome incidence	4,726,602	1,734,000	ONS Infection survey—30 Mar 2023	3,139,699

Table 2 shows the actual values of the data items that are still available and alternative entries chosen to replace, as near as possible, items that no longer are available. We now consider the key metrics in turn.

**Table 2:** Cumulative numbers of COVID-19 cases, deaths, tests, hospital admissions and post COVID-19 syndrome—for 12 months from 1st October 2023 to 30th September 2024 UK using November 2023 DCM with and without test & trace parameter change.

		DCM 2023 projection		Actual UK
Model with change in test trace parameter		Yes	No	
Outcomes	Data source of actual data	1st October 2023 to 30th September 2024		
Estimated incidence	Not available	40,692,662		Not available
Peak incidence per 100,000	Winter Covid-19 Infection Survey	494		538
Peak incidence date	Winter Covid-19 Infection Survey	29-Dec-23		19-Dec-23
Confirmed cases by PCR and LFT	UKHSA Dashboard	524,351	364,000	240,569
Deaths within 28 days of a positive PCR test	Not available	24,100	16,211	Not available
Deaths registered involved with COVID-19	ONS	14,940	9,857	14,023
Tests (both PCR and LFD)	Not available	14,080,675	13,439,509	Not available
PCR tests	UKHSA Dashboard	4,033,429	3,812,731	12,686,548
PCR pos tests	UKHSA Dashboard	306,967	290,171	1,245,849
Hospital admissions	UKHSA Dashboard	175,303	118,491	143,984
Post Covid-19 syndrome incidence	Winter Covid-19 Infection Survey	3,139,699	2,227,846	3,513,010

Alternative metrics have been chosen to replace those which are no longer available.

#### Incidence

**Figure 1:**
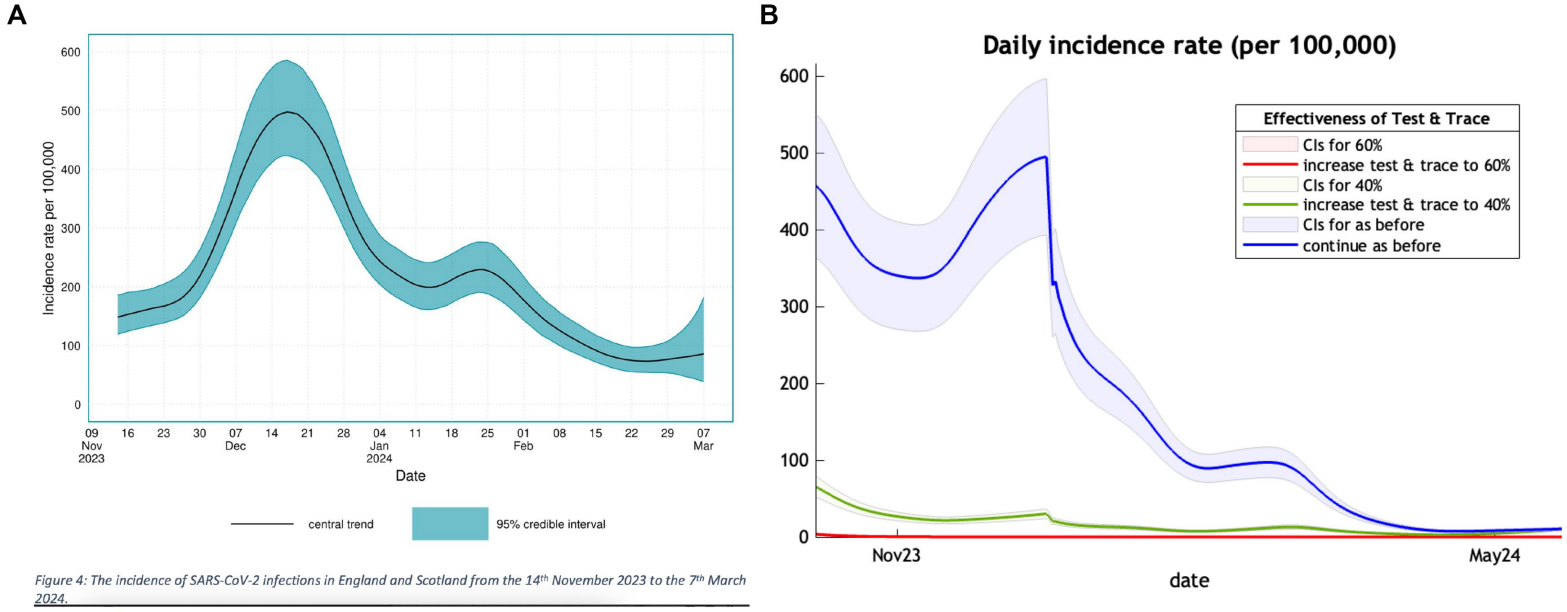
A comparison of the trend in incidence of new COVID-19 cases in the winter months of 2023 and 2024 between a UKSHA **(A)** (left panel) estimate and our projection **(B)** (right panel).

The Nov2023 model estimate of incidence in the twelve months suggested that 60% of the UK population would have had COVID-19 infection. The subsidiary analysis published by UKHSA provides a graph of their estimate of incidence in England and Scotland (Figure 1A). This suggests a peak incidence on 19 December 2023 of 498 cases, adjusted to 538 for the UK population. The corresponding DCM projection (Figure 1B) was remarkably consistent with a peak incidence of 494 on 29th December 2023.

The left panel of Figure 1 is adapted from Figure 4 of the UKHSA publication (21) showing their estimate of incidence of COVID-19 over the winter months of 2023/2024 with a peak in December. The right panel shows a section of Figure 1 of our previous publication (10) covering the same winter months with a similar peak in terms of value and timing.

#### Cases

Based on the Nov2023 model our estimate of confirmed cases is over double the number of actual cases counted by UKHSA. Free Lateral Flow Tests stopped being available, except to health staff and selected individuals on 1st April 2022. However, many people stopped testing themselves when their free test kits ran out or tested but did not report the result (25). This may explain the limited number of cases identified by UKHSA.

#### Deaths

The model has always estimated registered as well as deaths within 28 days of a positive PCR test and the cumulative number for the 12 months under study is shown in Table 2. The Nov2023 model over-estimated registered deaths involving COVID-19 by about 20%. This may be due to individuals—hospitalized with a positive COVID-19 test—who are likely to have COVID-19 as an incidental finding, rather than the primary reason for admission. In addition, those who die are usually registered with COVID-19 mentioned as involved in the death certificate (26). COVID-19 vaccination coverage had also changed for those over 64 years of age from 90% in January 2022 to 79% after the autumn booster program in January 2023 to 70% after the autumn booster program in January 2024. This could increase the chance of severe disease and death in this vulnerable age group.

#### PCR tests

Over the 4 years of the epidemic—for each confirmed case—there have been 6.5 positive PCR tests recorded. In the 12 months of the analysis there have been 7.2 tests to each case. The Nov2023 model underestimated the PCR test positive number four-fold. 1 in 10 PCR tests recorded on the UKHSA dashboard were positive as compared to the model projection of 1 in 13 PCR tests. The use of tests changed quite markedly in the later years.

#### Hospital admissions

The Nov2023 model over estimated hospital admissions by about 20%.

#### Long COVID

For the first time since the epidemic began, we can estimate with some confidence the incidence of Long COVID as we now have 4 years of experience of Long COVID and can estimate its average duration. Taking data from the Winter COVID-19 Infection Survey carried out by ONS in collaboration with UKHSA the average duration of Long COVID is 118 days. The prevalence found in March 2024 was 1.1 million individuals with post COVID-19 syndrome (symptoms of more than 12 weeks). The incidence was therefore 9,600 new cases per day or 3.5 million in the 12 months. This is similar to our projected value of 3.1 million provided by the Nov2023 model.

#### Summary of findings

The Nov2023 model offered projections which closely matched what transpired in respect of the incidence of cases and Long COVID; with predictions within 3 to 10% of yearly outcomes, respectively. The model projections of deaths and admissions were accurate to within about 20%. Projections of PCR tests were way off, plausibly due to changes in testing practice and availability. These findings provide a quantitative assessment of predictive accuracy that is consistent with previous reports applying similar analyses over the preceding years. These reports have used a prospective analysis comparing outcomes with predictions made in the past. We now consider a retrospective analysis—of the predictive validity of DCM—by evaluating the accuracy of predictions—based upon historical data—in relation to actual outcomes.

## Predictive accuracy of dynamic causal modeling

### Methods and data

To assess predictive accuracy over the entire pandemic, we used the model structure used in November 2023 and available on the DCM website (27). This model emerged via a process of Bayesian model selection as more data became available, and as reported in the literature. The priors over model parameters were based on expert consensus in the early phases of the epidemic. Posterior estimates of these parameters are based upon fitting the model to data. Because DCM is based upon a generative model, one can then use the posterior parameter estimates to generate data in the future. The ensuing projections are compared to what actually transpired, based on UKHSA and ONS data. The outcomes assessed were limited to those that were available throughout the reporting period. These outcomes are registered deaths involving COVID-19, cases confirmed by a positive PCR or LFT test, hospital admissions of patients with a positive test and Long COVID, defined as post COVID-19 syndrome (self-reported of more than twelve weeks duration). We focus on (i) peaks and troughs of incidence and (ii) cumulative values (area under the curve) to assess the overall morbidity and mortality predictions.

In brief, for each analysis of predictive accuracy, we estimated model parameters using data up until a reference point in time, under the same model structure. The resulting posterior estimates of the parameters were used to project three, six and 12 months into the future, to provide expected outcomes and their Bayesian credible intervals. We then assessed predictive accuracy in terms of when the empirical outcomes fall outside the Bayesian credible intervals. We report the results graphically for visual interpretation and in tabular format, by listing the percentage deviation between outcomes at each of the three time points in the future and their predictive posterior expectations. This procedure was repeated using data from the first, second, and third years, respectively. These yearly reference points were chosen as canonical stages in the pandemic; ranging from the second wave of the epidemic through to later stages foreshadowing endemic equilibrium. Specifically, the DCM were fitted to data from February 2020 to 30th September 2021, 2022, and 2023.

#### Data

Data sources are those used in the first section of this report. The incidence of Post COVID-19 syndrome was calculated using the findings of a global meta-analysis, with defined clusters of self-reported symptoms occurring 3 months after initial infection (28). This analysis found that the risk of long COVID—following symptoms—in the community is 7.9%, in hospital admissions is 27.9% and ARDS (acute respiratory distress syndrome) is 41.4%.

#### Priors

The Nov2023 DCM uses 60 parameters and calculates posterior parameters for each (Supplementary Table 1). These parameters are estimated under priors specified in terms of their mean and prior variance. Priors over seven illustrative parameters are listed in Table 3. For comparison, Table 3 also provides some recent empirical priors (with references), which were also evaluated (results not shown).

**Table 3:** Selected priors used in the models—original model parameters used in this analysis.

Parameters used in November 2023 DCM Covid model			Original model parameters						Recent empirical based priors					
Model code	Name	Description	Prior	Lower bound	Upper bound	Posterior	Lower bound	Upper bound	Prior	Lower bound	Upper bound	Posterior	Lower bound	Upper bound
tin	Infected period (days)	Latent period (between day infected and day infectious)	3	2.9	3.1	2.6	2.6	2.7	5.5	5.3	5.7	4.4	4.3	4.5
tcn	Infectious period (days)	Infectious period—presymptomatic and symptomatic infectious period	4	3.9	4.1	4.1	4.0	4.2	4.3	4.2	4.4	4.0	3.9	4.1
tim	Loss of natural immunity (days)	Loss of antibody immunity induced by Covid-19 infection	128	117.9	138.9	139.4	131.5	147.8	128	117.9	138.9	104.9	98.6	111.6
tic	Asymptomatic period (days)	Incubation period in days	4	3.7	4.3	2.2	2.1	2.2	6.5	6.3	6.7	5.1	4.9	5.2
tsy	Symptomatic period (days)	Symptomatic period in days	5	4.6	5.4	6.3	6.2	6.5	5	4.6	5.4	10.7	10.5	10.9
ttt	FTTI efficacy	Effectiveness of Find Test Trace Isolate system	0.036	0.029	0.045	0.036	0.029	0.045	0.25	0.20	0.31	0.29	0.26	0.32
iso	Self-isolation (days)	Days people isolate if a case or a known contact	8	7.8	8.2	8.7	8.4	8.9	6.7	6.5	6.9	7.4	7.1	7.6

References for this table: tin (31); tcn (32); tic (31); ttt (33) in line with international contact tracing with a successful tracing of 0.51 and assume HPTs have half this for non-household contacts 1/2/21; iso (34).

### Results

**Figure 2:**
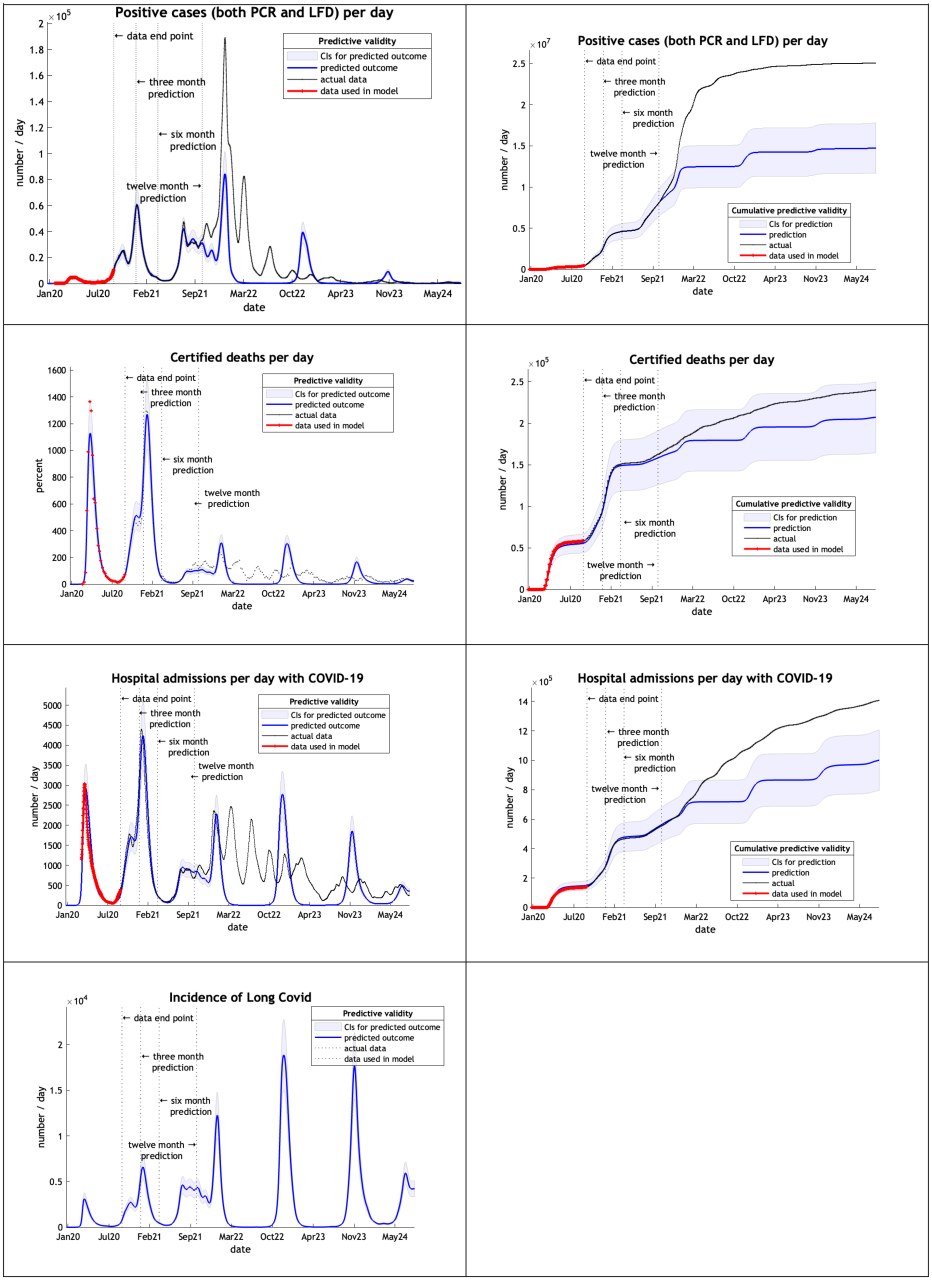
Model run using data from February 2020 to 30th September 2020.

With limited data of just 8 months from February 2020 to September 2020 the DCM provided very accurate predictions for up to 12 months into the future for positive cases, certified deaths and hospital admissions (Figure 2). Projections of certified deaths remained within the 90% credible intervals through to October 2024. This high level of predictive accuracy was not surprising and is consistent with an early *post-hoc* analysis described in the epilog of (4).

**Figure 3:**
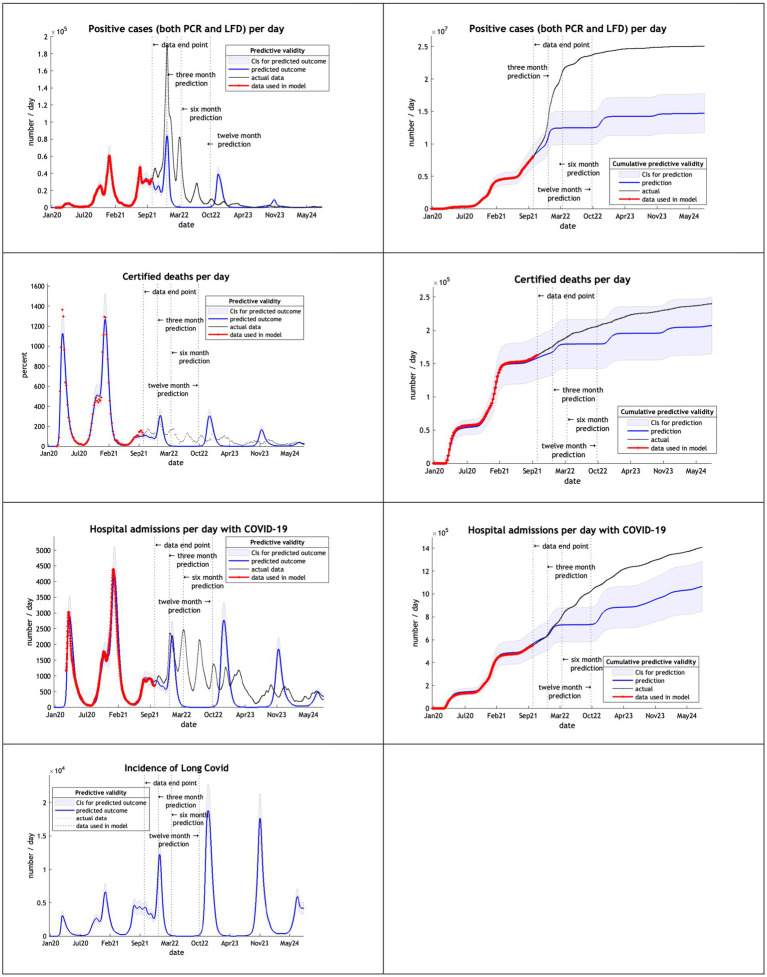
Model run using data from February 2020 to 30th September 2021.

Using 20 months of data, from February 2020 to September 2021, DCM predictions were reasonable only for 3 months (Figure 3). This might be due to the huge peak of cases in the winter months of 2020/2021 related to the delayed tightening of lockdown rules and, crucially, the arrival of the alpha variant.

**Figure 4:**
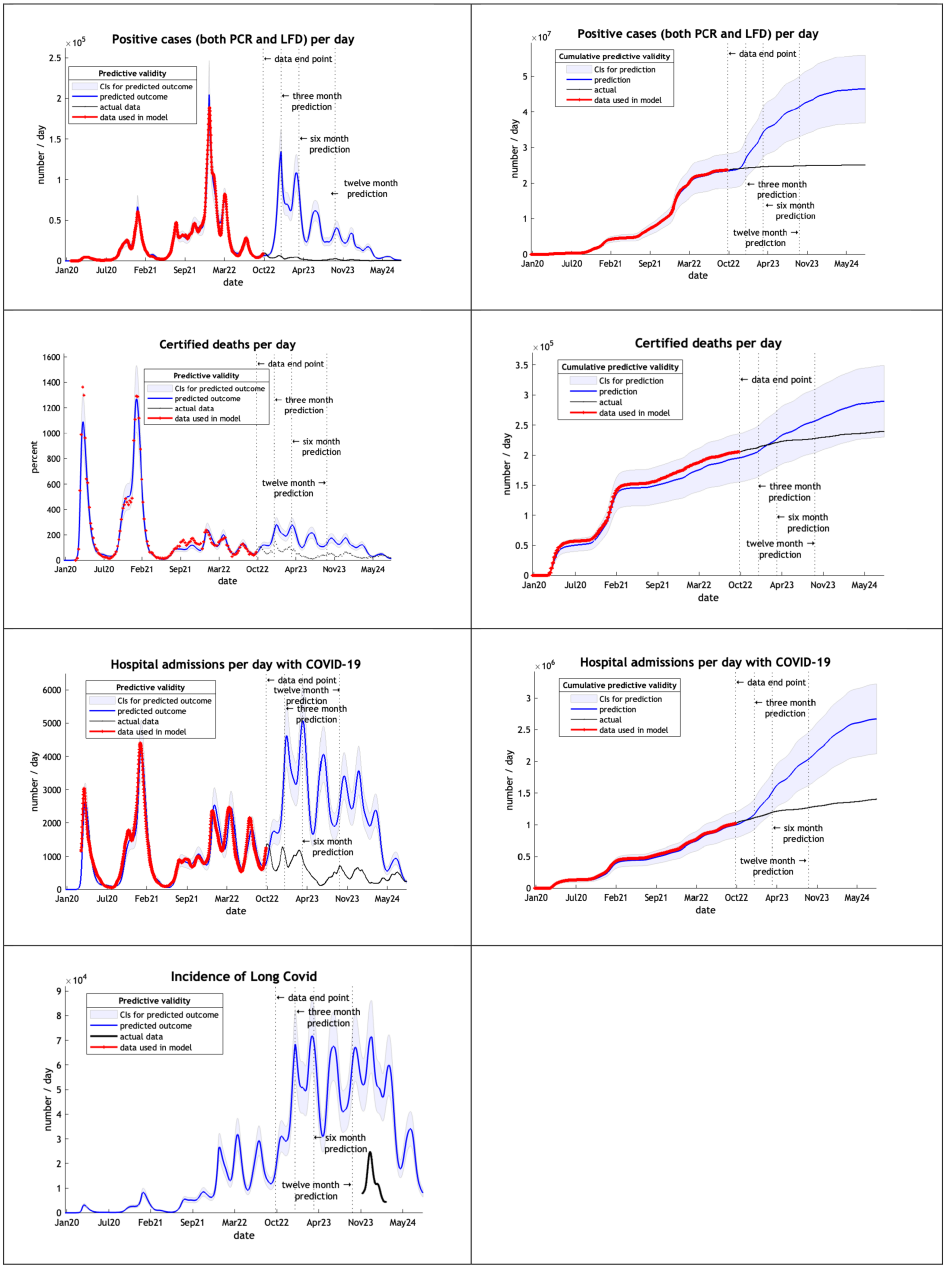
Model run using data from February 2020 to 30th September 2022.

The models, using data from February 2020 to September 2022, overestimated cases, deaths admissions and long covid after 3 months (Figure 4). Deaths remained within the credible interval range through to October 2024 and hospital admission for 6 months.

**Figure 5:**
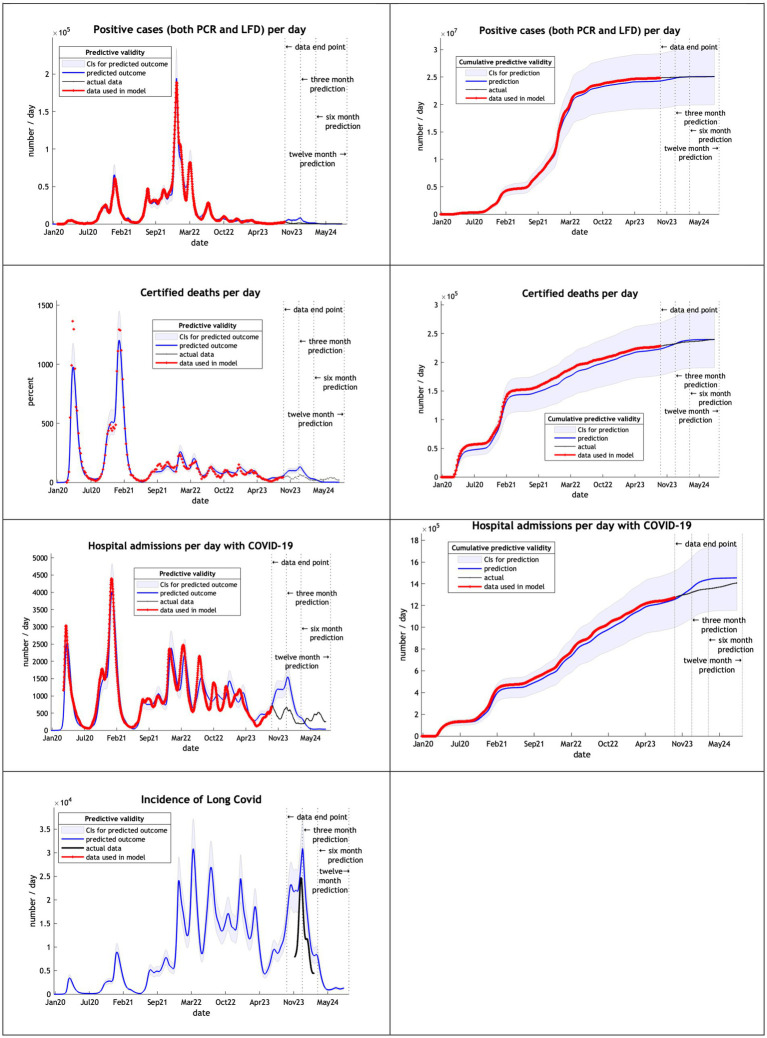
Model run using data from February 2020 to 30th September 2023.

The final model used data from the start of the epidemic to September 2023 (Figure 5). DCM accurately predicted cases and deaths, offering a reasonable hospital admissions estimate and a long covid projection within the credible interval of the UKHSA estimate of December 2023.

Note that in our graphical reporting we have provided predictions after the long term (12 month) forecast, up until a common endpoint (October 2024). In some cases, projections beyond 12 months are clearly implausible but illustrate some interesting features; namely, periodic fluctuations that speak to recurrent waves of infection that inherit from the interaction between epidemiological factors (e.g., prevalence) and sociobehavioural responses (e.g., self-isolation). However, for the purposes of the current analysis we operationally define long-term forecasts as forecasts at three, six and 12 months into the future—and do not consider projections beyond one year.

Table 4 shows the percentage deviation [(p-a)/a %] of the number of cases, deaths and hospital admissions predicted by the model (p) from the numbers which actually occurred from the start of the epidemic (a).

**Table 4:** The percentage deviation between the number of cases, deaths and hospital admissions at 3, 6, and 12 months and their cumulative predictive posterior expectations each year since 1st February 2020.

Model and outcome measures	Percentage deviation from actual		
	Projected 3 months	Projected 6 months	Projected 12 months
Model run from 1st October 2020			
Positive cases (both PCR and LFD) per day	3.9%	1.9%	5.2%
Certified deaths per day	3.1%	2.3%	3.3%
Hospital admissions per day with COVID-19	−1.0%	−0.5%	1.4%
Model run from 1st October 2021			
Positive cases (both PCR and LFD) per day	12.8%	39.0%	50.3%
Certified deaths per day	5.4%	6.1%	14.5%
Hospital admissions per day with COVID-19	1.8%	8.0%	31.1%
Model run from 1st October 2022			
Positive cases (both PCR and LFD) per day	0.0%	−27.3%	−63.5%
Certified deaths per day	4.2%	−0.8%	−11.7%
Hospital admissions per day with COVID-19	−0.8%	−19.2%	−57.3%
Model run from 1st October 2023			
Positive cases (both PCR and LFD) per day	13.9%	14.0%	14.2%
Certified deaths per day	14.1%	15.4%	17.1%
Hospital admissions per day with COVID-19	23.9%	25.9%	28.8%

#### Summary

In the first year of the analysis from February to September 2020 the model provided very accurate predictions at three, six and twelve months (i.e., to within 5%). Furthermore, the actual outcomes for all key metrics considered were comfortably within the 90% Bayesian credible intervals. As noted above, this is consistent with early experience with the model during the first phase of the pandemic, prior to the emergence of highly transmissible variants. In the second year of the analysis from February 2020 until September 2021, the projections retained reasonable accuracy for three-month forecasts (less than 13% deviation) but were less accurate thereafter; on occasion actual outcomes transcending the Bayesian confidence intervals at six and 12 months in the future.

By the third year—using estimates based upon data from February 2020 until September 2022—the model offers reasonable estimates for three months but not at six or twelve months. This may well relate to the emergence of the Omicron variants and changes in population behavior at that time. In the final year the model based upon data from February 2020 until September 2023 appeared to regain predictive accuracy, in the sense that cumulative cases, deaths and hospital admissions were predicted accurately with 12-month predictions all less than 30% deviations and well within the Bayesian confidence intervals.

## Discussion

In summary, the prospective and, in particular, retrospective analyses speak to a nontrivial predictive validity of the dynamic causal model used for forecasting, nowcasting and scenario modeling during the recent COVID pandemic. In brief, key metrics such as daily deaths, hospital admissions and morbidity—reflected in the incidence of Long COVID—can generally be predicted with about a 20% accuracy, one year into the future. A more fine-grained (retrospective) analysis suggests that the long-term forecasting over 3 and 6 months can be remarkably accurate under certain conditions. The current analysis suggests that in the early phases of the epidemic—before the arrival of highly transmissible strains of the coronavirus—predictive accuracy is remarkably high, in many cases less than 5% deviation. This high accuracy re-emerges toward the end of the pandemic, when there is sufficient data to estimate parameters controlling slow fluctuations in factors that control viral spread. In the DCM, these factors include things like seasonal variations and slow declines in virulence, set against progressive but diminishing increases in transmissibility.

This speaks to an important trade-off among the factors that determine predictive validity. Put simply, in the early phases of the pandemic, slow fluctuations in transmissibility and virulence have yet to be expressed and therefore do not confound model predictions. However, there is less data available to provide precise estimates of key epidemiological and sociobehavioural model parameters, which are necessary for confident and accurate predictions. Conversely, when the available timeseries covers several years, more precise estimates of changes in transmissibility, virulence, and long-term sociobehavioural responses can be estimated.

It could be argued that the recent coronavirus pandemic represents an unprecedented opportunity for complex system modeling, in the sense that there is a wealth of data to constrain model selection and the parameterisation of selected models. In a similar vein, this means that the opportunity for assessing the predictive validity of different models is itself unprecedented. This may explain why the current report cannot refer to any comparative analyses of predictive validity in epidemiology; largely, because there are none. This may also reflect the fact that dynamic causal modeling is in a unique position to offer long-term forecasts due to its construction: unlike other forms of epidemiological modeling, dynamic causal models come equipped with a reliable measure of their quality in the form of (variational bounds on) model evidence or marginal likelihood. This means that one can compare different models; thereby not just optimizing the parameters of any given model but optimizing the structure and expressiveness of these models as data is assimilated (5).

This casts our retrospective analysis of predictive validity in a certain light: we have used a model that was selected over a four-year period to model data generated prior to model selection. This might explain the high levels of predictive accuracy when compared to conventional models; e.g. (7). A detailed description of the differences between the DCM and conventional epidemiological models can be found in the foundational papers introducing the DCM for COVID: e.g. (8). In brief, the DCM can be regarded as an extension of conventional (SEIR) models to incorporate sociobehavioural responses—at the population level—that enable the model to generate a wide variety of timeseries data; ranging from data reporting the prevalence and pathogenicity through to measures of socio-economic activity. The ensuing generative model allows one to identify the latent states that best explain viral spread through Bayesian model inversion and subsequent model comparison. Crucially, DCM uses variational procedures for model inversion—thereby eschewing sampling schemes such as Monte Carlo Markov chain—and, crucially, furnishing a measure of model evidence (a.k.a., marginal likelihood) in terms of a variational free energy bound on log evidence. This enables the model to be updated via Bayesian model selection, as new data become available; a process that was documented in the literature and at the following website (29).^1^

The DCM has been used for modeling the pandemic in other countries, with published results (3, 6, 13, 29). In terms of predictive validity, we could find only one published study. This report used the median absolute percent error (MAPE) scores of conventional model forecasts to three months (30). The study compared the models of six institutions and found MAPE results looking at cumulative deaths for the same period considered in our analysis (Table 4), starting in October 2020 and looking forward 3 months. They reported an average of 32% MAPE ranging from 45 to 18%. This compares to our MAPE of 3.1%, which represents an order of magnitude improvement in predictive accuracy. However, this comparison should not be overinterpreted, as our model was more mature and expressive, and looked at the UK only (as opposed to their estimates, based on a composite score of high-income countries).”

However, our results beg the question whether this is a useful assessment. The answer to this question depends upon whether the next pandemic can be explained under the structure identified using data from the previous pandemic. Clearly, one cannot know this in advance. However, having the current model in place [with its reduced variants (30)] means that one can assess its suitability using the model evidence or marginal likelihood of data from the next pandemic. If the DCM described in this, and the supporting, literature is apt, then one would anticipate a similar predictive validity described above.

One could argue that it is important to establish the predictive validity of this kind of modeling—using the retrospective analysis presented above—for future deployment of DCM in other outbreaks, or application domains (e.g., climate change, food insecurity, *et cetera*). In principle, one could use the current structure of the DCM—and accompanying posterior estimates—as priors for a subsequent outbreak. The advantage of having a valid generative model of this sort is that one can rollout into the future and predict what would happen under different scenarios or interventions. This was one of the motivations for the current application of DCM to help with situational awareness (e.g., nowcasting) and, more importantly, help decision-making through scenario modeling equipped with uncertainty quantification.

Another possible advantage of the use of a DCM model is the wide range of parameters optimized that could be used to provide estimates for use in other models and studies. The current model used 60 parameters which together generate the outcomes shown in the figures and tables. Infectious disease epidemiology research units may wish to add the DCM to their portfolio of models, to provide estimates of these parameters that could be incorporated into other models.

## Data Availability

Publicly available datasets were analyzed in this study. This data can be found at: https://www.fil.ion.ucl.ac.uk/spm/covid-19/.

## Footnotes

1 Technically, the variational procedure used for model inversion—i.e., optimising posteriors over model parameters determining the transitions among different states of infection, symptomatology, location, *et cetera*—is called variational Laplace. This provides an analytic form of approximate Bayesian inference—using natural gradient descent—that is afforded by assuming a functional form for the (approximate) posteriors over model parameters. This functional form was a Gaussian density, specified in terms of posterior expectations and covariances; hence, variational Laplace.
