# Correction: Dynamic causal models in infectious disease epidemiology—an assessment of their predictive validity based on the COVID-19 epidemic in the UK 2020 to 2024

**DOI:** 10.3389/fpubh.2025.1766577

**Published:** 2026-01-30

**Authors:** Cam Bowie, Karl Friston

**Affiliations:** 1 Retired, Devon, United Kingdom; 2 Queen Square Institute of Neurology, University College London, London, United Kingdom

**Keywords:** coronavirus, compartmental models, epidemiology-descriptive, incidence, public health methodology, dynamic causal modeling

The reference for 30 was erroneously written as “Friston K, Parr T, Zeidman P. Bayesian model reduction. *arXiv*. (2019). doi: 10.48550/arXiv.1805.07092”. It should be:

“Friedman J, Liu P, Troeger CE, Carter A, Reiner RC, Barber RM, et al. Predictive performance of international COVID-19 mortality forecasting models. *Nat Commun*. (2021) 12:2609. doi: 10.1038/s41467-021-22457-w.”

The original version of this article has been updated.

