# Supplementary material for: Dynamic causal models in infectious disease epidemiology—an assessment of their predictive validity based on the COVID-19 epidemic in the UK 2020 to 2024
Source: Front Public Health. 2025 May 2;13:1573783. doi: 10.3389/fpubh.2025.1573783 (PMC12081349; doi:10.3389/fpubh.2025.1573783)
Supplement: Supplementary file 1 [file Data_Sheet_1.PDF]

The parameters used in the DCM model using priors (column d) based on expert consensus in the early phase of the epidemic

Posterior parameter values (column h) identified by the model as those used in a generative model that best fit the data series available such as registered deaths, positive tested cases and hospital admissions

number	name	description	prior	precision	lower	upper	posterior	lower bound	upper bound
a	b	c	d	e	f	g	h	i	j
1	n	population size (M)	12.0065	Inf	12.0065	12.0065	12.0065	12.0065	12.0065
2	n	initial cases	8.39E-05	0.135335	9.59E-07	0.00733485	2.41E-08	1.85E-08	3.15E-08
3	r	pre-existing immunity	0.1	403.429	0.0921371	0.108534	0.0776071	0.0716583	0.0840498
4	o	initially exposed	0.1	54.5982	0.080043	0.124933	0.0490707	0.039415	0.0610919
5	m	relative eflux	0.1	54.5982	0.080043	0.124933	0.056997	0.0457767	0.0709674
6	out	P(leaving home)	0.4	403.429	0.368548	0.434136	0.282951	0.269251	0.297349
7	sde	time constant of lockdown	4	54.5982	3.20172	4.99732	4.15001	3.32438	5.18071
8	qua	time constant of unlockin	128	403.429	117.935	138.923	328.16	319.982	336.546
9	exp	viral spreading (days)	0.02	54.5982	0.0160086	0.0249866	0.0236958	0.022031	0.0254863
10	hos	admission rate (hospital)	2	54.5982	1.60086	2.49866	2.28813	1.83159	2.85846
11	ccu	admission rate (critical)	0.2	54.5982	0.160086	0.249866	0.236005	0.200726	0.277484
12	s	infectivity changes	1	403.429	0.921371	1.08534	1.21437	1.11982	1.31689
13	nin	contacts: home	1	403.429	0.921371	1.08534	1.05658	0.986126	1.13207
14	nou	contacts: work	16	403.429	14.7419	17.3654	25.7203	24.4712	27.0331
15	trn	transmission strength	0.2	403.429	0.184274	0.217068	0.272342	0.264309	0.280618
16	trm	seasonal transmission	0.04	54.5982	0.0320172	0.0499732	0.0242303	0.0203608	0.0288352
17	tin	infected period (days)	3	2980.96	2.91097	3.09175	2.61755	2.55193	2.68486
18	tcn	infectious period (days)	4	2980.96	3.88129	4.12234	4.14083	4.03988	4.24429
19	tim	loss of natural immunity (days)	128	403.429	117.935	138.923	139.403	131.506	147.773
20	res	resistance	0.2	403.429	0.184274	0.217068	0.158334	0.146751	0.17083
21	tic	asymptomatic period (days)	4	403.429	3.68548	4.34136	2.16366	2.08363	2.24677
22	tsy	symptomatic period (days)	5	403.429	4.60686	5.4267	6.31656	6.16726	6.46948
23	trd	critical period (days)	16	54.5982	12.8069	19.9893	10.558	10.4358	10.6816
24	sev	P(ARDS|symptoms): initial	0.002	7.38906	0.00109203	0.0036629	0.00699639	0.00667502	0.00733322
25	lat	P(ARDS|symptoms): change	1	7.38906	0.546016	1.83145	1.77068	1.73194	1.8103
26	fat	P(fatality|ARDS): initial	1.00E-06	7.38906	5.46E-07	1.83E-06	1.00E-06	5.46E-07	1.83E-06
27	sur	P(fatality|ARDS): change	1	7.38906	0.546016	1.83145	2.3826	2.3027	2.46528
28	ttt	FTTI efficacy	0.036	54.5982	0.0288155	0.0449758	0.0362809	0.0291181	0.0452057
29	tes	testing: bias (PCR)	16	7.38906	8.73625	29.3032	8.43504	7.83196	9.08455
30	tts	testing: bias (LFD)	1	7.38906	0.546016	1.83145	1.4235	0.78536	2.58015
31	del	test delay (days)	3	403.429	2.76411	3.25602	2.91064	2.70172	3.13573
32	vac	vaccine seroconversion (days)	32	403.429	29.4839	34.7309	32.8621	30.5549	35.3436
33	fnr	false-negative rate	0.08	403.429	0.0737097	0.0868271	0.0519395	0.047895	0.0563255
34	fpr	false-positive rate	0.0002	403.429	0.00018427	0.00021707	0.00021509	0.00019833	0.00023326
35	lim	testing: capacity	0.0005	7.38906	0.00027301	0.00091573	0.00120871	0.00110935	0.00131696
36	rat	testing: constant	8	403.429	7.37097	8.68271	8.39729	7.75202	9.09627
37	ons	testing: onset	100	0.135335	1.14339	8745.95	193.364	190.285	196.492
38	lag	reporting lag	1	Inf	1	1	1	1	1
39	inn	seasonal phase	1	7.38906	0.546016	1.83145	790.227	585.164	1067.15
40	mem	vaccination rollout (days)	128	2980.96	124.201	131.915	125.532	122.435	128.707
41	rol	vaccination rollout (1st)	0.0001	16	6.63E-05	0.00015087	0.00010361	6.87E-05	0.0001563
42	fol	vaccination rollout (2nd)	0.0001	16	6.63E-05	0.00015087	8.77E-05	5.82E-05	0.00013196
43	vef	vaccine efficacy: sterilising	0.4	403.429	0.368548	0.434136	0.746056	0.708524	0.785577
44	lnk	vaccine efficacy: pathogenicity	0.24	Inf	0.24	0.24	0.24	0.24	0.24
45	ves	vaccine efficacy: transmission	0.1	403.429	0.0921371	0.108534	0.486256	0.466711	0.50662
46	lnf	vaccine efficacy: fatality	0.05	403.429	0.0460686	0.054267	0.173085	0.163745	0.182957
47	con	LFD confirmation	0.2	7.38906	0.109203	0.36629	0.0988878	0.056595	0.172786
48	iso	self-isolation (days)	8	2980.96	7.76258	8.24468	8.68744	8.43279	8.94978
49	tnn	loss of T-cell immunity	256	2980.96	248.403	263.83	268.716	261.358	276.282
50	lnr	LFD specificity	0.46	403.429	0.423831	0.499256	0.914871	0.912148	0.917601
51	lpr	LFD sensitivity	0.0002	403.429	0.00018427	0.00021707	0.00019042	0.00017552	0.00020658
52	rel	PCR testing of fatalities	1	54.5982	0.80043	1.24933	0.997157	0.930175	1.06896
53	pro	contact rate decay (days)	1	54.5982	0.80043	1.24933	11.8567	11.5308	12.1918
54	oth	survival risk in care homes	0.1	54.5982	0.080043	0.124933	0.0810612	0.0650124	0.101072
55	iad	changes in transfer to CCU	1	7.38906	0.546016	1.83145	1.52475	1.46786	1.58384
56	tra	transmissibility parameters	0.08	403.429	0.0737097	0.0868271	0.0498244	0.046059	0.0538977
57	dps	doses per seroconversion	2	403.429	1.84274	2.17068	2.28605	2.24609	2.32672
58	abs	age-related testing	1	403.429	0.921371	1.08534	0.984561	0.90967	1.06562
59	iss	self-isolation	1	403.429	0.921371	1.08534	1.6064	1.52456	1.69262
60	rut	Sensitivity to contact rate	1	403.429	0.921371	1.08534	0.468567	0.439942	0.499054
